# Dynamic Patterns of Forces and Loading Rate in Runners with Unilateral Plantar Fasciitis: A Cross-Sectional Study

**DOI:** 10.1371/journal.pone.0136971

**Published:** 2015-09-16

**Authors:** Ana Paula Ribeiro, Silvia Maria Amado João, Roberto Casanova Dinato, Vitor Daniel Tessutti, Isabel Camargo Neves Sacco

**Affiliations:** 1 University of Sao Paulo, Physical Therapy, Speech and Occupational Therapy Department, School of Medicine, São Paulo, Brazil; 2 University of Santo Amaro, Physical Therapy Department, School of Medicine, São Paulo, Brazil; University of Bath, UNITED KINGDOM

## Abstract

**Aim/Hypothesis:**

The etiology of plantar fasciitis (PF) has been related to several risk factors, but the magnitude of the plantar load is the most commonly described factor. Although PF is the third most-common injury in runners, only two studies have investigated this factor in runners, and their results are still inconclusive regarding the injury stage.

**Objective:**

Analyze and compare the plantar loads and vertical loading rate during running of runners in the acute stage of PF to those in the chronic stage of the injury in relation to healthy runners.

**Methods:**

Forty-five runners with unilateral PF (30 acute and 15 chronic) and 30 healthy control runners were evaluated while running at 12 km/h for 40 meters wearing standardized running shoes and Pedar-X insoles. The contact area and time, maximum force, and force-time integral over the rearfoot, midfoot, and forefoot were recorded and the loading rate (20–80% of the first vertical peak) was calculated. Groups were compared by ANOVAs (*p*<0.05).

**Results:**

Maximum force and force-time integral over the rearfoot and the loading rate was higher in runners with PF (acute and chronic) compared with controls (p<0.01). Runners with PF in the acute stage showed lower loading rate and maximum force over the rearfoot compared to runners in the chronic stage (p<0.01).

**Conclusion:**

Runners with PF showed different dynamic patterns of plantar loads during running over the rearfoot area depending on the injury stage (acute or chronic). In the acute stage of PF, runners presented lower loading rate and forces over the rearfoot, possibly due to dynamic mechanisms related to pain protection of the calcaneal area.

## Introduction

Running is one of the most popular sport activities worldwide, since it is available for all ages at a low cost, and is versatile and associated with health benefits [1–3]. The prevalence of lower limb injuries has risen with running’s increased popularity over the last 30 years [1,2], among which plantar fasciitis (PF) is one of the most prevalent [3, 4].

Plantar fasciitis is a musculoskeletal disorder characterized by localized pain on the plantar fascia insertion, which is exacerbated in the mornings after getting up or after long rest periods [5, 6, 7]. Although there are several intrinsic and extrinsic factors related to the development of PF [5], some have drawn more attention in both clinical and research settings, such as longitudinal plantar arch alterations [4, 6, 7], rearfoot pronation [8], and magnitude of plantar loads [8–11].

Among all of the factors, plantar loads over the calcaneal area have been described as one of the primary risk factors for PF development [12–14]. Excessive loads promote stretching of the plantar fascia, which stimulates microtraumas and subsequent changes in the connective tissues, which in turn initiates an acute inflammatory response with fibroblast proliferation [15–17]. The repetitive impact of the heel can result in a chronic process, followed by degeneration and fragmentation of the plantar fascia and by fibrosis formation without inflammatory response in the medial calcaneal tuberosity [17, 18].

Because PF is the third most-common injury in runners [3, 4], one would expect that this motor task—running—would be the focus of studies that investigate the risk factors of PF development in the population. However, the majority of studies addressing PF biomechanical issues have investigated the effect of plantar loads during walking in non-athletes with plantar fasciitis [9–11]. In particular, these walking studies have observed that the pain stimulus in the feet of individuals with PF (inflammatory stage) promoted changes in plantar loads during the support phase of walking. These changes resulted in higher loads over the more anterior parts of the foot, such as the midfoot [11], forefoot [9], and toes [10], but not over the calcaneal area (rearfoot region), as expected in the pathophysiology development of PF [8–10].

According to Wearing *et al*. (2007) [11], symptomatic feet make some adaptations during gait to reduce the load on the rearfoot. This study proposed two possible theories. First, it is not possible to infer if increased loads in other areas of the foot, such as the midfoot and forefoot, as described by some authors, in fact contribute to the development of plantar fasciitis by inducing the stretching of the fascia and increasing the tension stress on its insertion into the medial tuberosity of the calcaneus. Second, it is also not possible to infer whether the presence of pain in the rearfoot would promote protective mechanisms, which could reduce the plantar load in this area [11], or whether these anterior loads are a contributing factor to the development of PF.

Therefore, it is important to investigate whether even in the absence of pain, during the chronic stage of PF, the dynamic load distribution pattern observed while walking in the acute stage of PF is similar to that during running. Recently, the effects of the different stages of PF and the presence or absence of pain have been studied during running [6, 8]; however their findings were contradictory. The first study showed that women with a history of PF (in the chronic stage of PF), without the presence of pain, showed higher loading rates compared to controls [6]. This study did not include runners with PF and presence of pain. The second study found that runners with acute PF (with pain) and chronic PF (without pain) had a similar dynamic pressure distribution pattern in comparison to controls [8]. The difference in the results of both studies may be due to the variables chosen to represent loads and to the environment used to evaluate the runners. The first study used the vertical ground reaction force from a force plate in a laboratory environment [6], whereas the second used plantar pressure variables from instrumented insoles and a running track for training and competition [8].

The importance of studying the dynamic plantar loads in natural environments for training and competition was recently highlighted by Hong *et al*. (2012) [19], who found that the distribution of these loads during running on a treadmill were not the same as those observed during running on fixed ground surfaces. According to these authors, running on treadmills could even be employed in rehabilitation programs to help reduce plantar loads. However, for individuals with lower limb injuries, the research has shifted the paradigm from the treadmill to the ground, as a more ecologically valid environment is crucial to better understand the causal factors involved in the runners’ daily routines. Because running is a cyclic modality, whose impacts on the heel, plantar ligaments and plantar fascia are of great magnitude (3.7 to 4.8 times the body weight) [20], its continuous practice could be directly related to the onset and progression of PF. A better understanding of the plantar load patterns during running in natural environments could lead to better therapeutic benefits and better-designed rehabilitation programs for lower limb injuries, such as PF.

The purpose of this study was to analyze and compare the dynamic patterns of plantar loads over foot areas and the loading rate during running of runners with acute-stage PF to those in the chronic stage of the injury in relationship to healthy runners. The hypotheses of the study were: (1) runners with both acute and chronic PF would show higher dynamic plantar loads over the rearfoot, compared to controls; (2) runners in the acute stage of PF would have lower levels of dynamic plantar loads over the rearfoot compared to runners in the chronic stage; and (3) runners in the acute stage would present higher plantar loads over the midfoot and forefoot, and lower loads over the rearfoot, due to pain caused by inflammation.

## Materials and Methods

### Participants

Seventy-five recreational runners of both sexes (45 with PF and 30 healthy controls) were recruited by specific electronic media related to running activities and from the Rehabilitation Center of Sport Rheumatology of the University Hospital of São Paulo, Brazil. The mean running speed of their last 10-km competition was 11.7± 0.6 km/h, as reported by the subjects. For inclusion in this study, the runners had to: have run at least 20km weekly for at least one year; be experienced in long-distance competitions; have a rearfoot strike pattern; have had no history of prior surgery, traumas, or fractures of the lower limbs in the prior six months; have a maximum leg length discrepancy of 1cm; and had no other musculoskeletal disorders, such as neuropathies, obesity, rheumatoid arthritis, or calcaneus spurs. All participants provided written consent, based upon ethical approval by the Human Research Board of the School of Medicine, University of São Paulo (approval the protocol of research, number: 384/10; title: Support standard and impact of the feet with the ground during the running of runners with history and symptoms of plantar fasciitis and its relationship to the medial longitudinal arch).

All 45 runners had a clinical diagnosis of unilateral PF, which was confirmed by ultrasonography [21] to better differentiate between the different stages of the injury. Thirty runners showed inflammatory processes in the ultrasound (hypoechoic changes, perifascial fluid collection, and fibroblast proliferation), and were considered in the acute stage of the injury (acute PF group). They presented pain symptoms over the heel for more than four months (mean of 5.3±2.2 months), with an intensity of 7.8cm, assessed by means of a visual analog scale. The pain was present during palpation of the plantar fascia, after waking up in the mornings, while remaining in the standing position, or when performing the first steps of walking, as well as while maintaining long periods in a static standing position or sitting position, and after physical activities of short duration [11, 22].

Fifteen runners with unilateral PF showed plantar fascia thickness, fragmentation, and degeneration in the ultrasound, but no signs of acute inflammatory processes [17]. They had a mean diagnosis time of 1.5±3.3 years and were pain-free for more than two months. These runners were considered in the chronic stage of the injury (chronic PF group).

No differences among groups were found for demographic and anthropometric characteristics, as demonstrated in Table 1.

**Table 1:** Descriptive statistics (mean ± standard deviation) and comparisons between acute plantar fasciitis (PF), chronic PF and control groups regarding their demographic, anthropometric, and running practice characteristics.

Variables	Acute PF (1)	Chronic PF (2)	Controls (3)	p^&^
Age (years)	45.4±8.1	38.3±3.3	37.0±2.0	0.191
Sex (%)	F (40); M (60)	F (36,6); M (63,4)	F (36,6); M (63,4)	-
Body mass (kg)	69.6±14.0	72.3±10.0	60.5±5.0	0.585
Height (m)	1.68±9.2	1.76±7.8	1.74±7.0	0.173
Body mass index (kg/m^2^)	24.3±2.9	23.0±2.0	25.5±2.0	0.307
Training volume (Km/week)	40.0±12.0	45.0±10.0	42.0±8.5	0.110
Practice time (years)	7.0±5.0	6.2±5.0	5.1±3.8	0.140

^*&*^ *p* value were calculated using *one-way ANOVAs*.

### Procedures and Instruments for the Assessment of Plantar Pressures

The plantar pressure distributions were obtained during running with insoles of the Pedar-X system (Novel, Munich, Germany) at frequencies of 100Hz. All runners wore standardized running shoes (Rainha System, Rainha, Alpargatas, São Paulo, Brazil, USA sizes 7–12). The shoe characteristics included an insole made by ethylene vinyl acetate (EVA with compression set: 56%, hardness: 57 Asker C and density = 0.21 g/cm3) throughout the entire shoe sole, composed of light and highly resilient plastic that disperses the impact horizontally before returning quickly to the initial state. It is recommended by manufacturers for those seeking a running shoe with a neutral strike. The instrumented insoles were placed between the socks and the shoes and were connected to the 1.5-kg equipment inside a backpack [8]. The insoles were 2.5mm thick and contained a matrix of 99 capacitive pressure sensors with a spatial resolution of 1.6 to 2.2cm^2^.

The runners underwent a pre-trial adaptation phase, using the required footwear and the backpack with the equipment. Subjects ran a distance of 40 meters on a regular asphalt surface at 12 km/h ±5%km/h. The runners were considered adapted to the environment (backpack and shoes) when the mean speed of three consecutive trials over 40 meters was 12 km/h±5% [8, 23]. Two observers used a digital stopwatch to control the speed simultaneously, within the central 20 meters. The inter-rater agreement was found to be excellent, with an intra-class correlation coefficient of 0.96.

Approximately 30 steps were acquired and the variables of interest were calculated using a custom-written MATLAB function. The mean value of the 30 steps per subject was used for statistical purposes. The contact area (cm^2^), contact time (ms), maximum force (times body weight), and force-time integral (times body weight.ms) over the rearfoot, midfoot, and forefoot were recorded. The force data were analyzed by a MATLAB routine and normalized by the body weight (BW). The plantar loading rate was calculated from the vertical force; loading rate was 80% [BW.s^-1^], defined as the force rate between 20% and 80% of the contact time from heel strike to the first vertical peak.

### Statistical Analyses

The sample size calculation of the 75 runners was based upon the maximal force variable, was carried out using G-Power 3.0 software, and considered a moderate effect size (*F* = 0.25), a power of 80%, and a significance level of 5%.

All outcome measures showed normal distributions (Shapiro-Wilk test) and homogeneity of variances (Levene’s test). For the control group, force data of one foot per subject was randomly selected for statistical comparisons with the PF groups (acute and chronic). For the PF groups, force data from the affected foot (unilateral PF) was analyzed and compared to the other groups. One-way ANOVAs followed by Newman-Keuls post-hoc tests were employed to compare groups regarding the anthropometric, demographic, and running practice characteristics and plantar loading rate. Groups and plantar areas were compared using two-way ANOVAs for repeated measures (3 groups × 3 plantar areas) for force and contact area/time variables, followed by Newman-Keuls post-hoc tests. To describe the effect size between studied groups, the Cohen’s d coefficients were calculated. All analyses were carried out with Statistica software (version 7.0). We adopted a significance level of 5%.

## Results

The plantar loading rate of 20–80% (*F* = 7.16, DF = 2, *p* = 0.001) was higher in both PF groups compared to the controls, with effect sizes from moderate to large. The high plantar load rate (20–80%), with large effect sizes, was observed in the chronic PF group compared to controls. Runners with PF in the acute stage showed lower loading rates compared to runners in the chronic phase, with a moderate effect size (Table 2).

**Table 2:** Descriptive statistics (mean ± standard deviation) and comparisons between acute plantar fasciitis (PF), chronic PF and control groups regarding the plantar loading rate normalized by body weight (BW).

Variable	Acute PF (1)	Chronic PF (2)	Controls (3)	p-values^&^	Effect size (Cohen`s d)
Loading rate (20–80%) (BW/s)	0.76±0.20	0.89±0.27	0.64±0.16	0.047 ^(1–2)^	0.59 ^(1–2)^ (medium)
				0.001 ^(1–3)^	0.67 ^(1–3)^ (medium)
				0.034 ^(2–3)^	1.26 ^(2–3)^ (large)

^*&*^ *p* value were calculated using ANOVAS test and *Newman-Keuls post-hoc test*.

The maximum force (*F* = 3.81, DF = 4, *p* = 0.005), force-time integral (*F* = 2.70, DF = 4, *p* = 0.047), and contact area over the rearfoot (*F* = 9.10, DF = 4, *p* = 0.002) were higher in runners with PF (acute and chronic) compared to controls, with effect sizes ranging from moderate to large (Table 3). The contact time over the rearfoot (F = 2.75, DF = 4, *p* = 0.212), midfoot (F = 6.27, DF = 4, *p* = 0.082) and forefoot (F = 1.23, DF = 4, *p* = 0.245) were similar among the acute- and chronic-stage PF and control groups. Runners with PF in the acute stage showed lower maximum force over the rearfoot compared to runners in the chronic stage, with a small effect size (Table 3). The total contact time over the foot (F = 0.55, DF = 4, *p* = 0.867) also was similar among the acute- and chronic-stage PF and control groups, as shown in Table 4.

**Table 3:** Descriptive statistics (mean ± standard deviation) and comparisons between acute plantar fasciitis (PF), chronic PF and control groups regarding their maximum force and force-time integral (normalized by body weight, BW) and contact area in each plantar area.

Variables	Groups	Rearfoot	Midfoot	Forefoot	Effect size (Cohen´s d) rearfoot
**Maximum force (BW)**	Acute PF (1)	1.34 ±0.29	0.55±0.17	1.73±0.48	0.30 ^(1–2)^ (small)
	Chronic PF (2)	1.46 ±0.46	0.40±0.09	1.31±0.30	0.64 ^(1–3)^ (medium)
	Control (3)	1.19±0.17	0.46±0.10	1.49±0.21	0.93 ^(2–3)^ (large)
	***p*** ^&^ ***-value***	*0*.*020* ^*(1–2)*^	*> 0*.*05*	*> 0*.*05*	
		*0*.*029* ^*(1–3)*^	*> 0*.*05*	*> 0*.*05*	
		*0*.*001* ^*(2–3)*^	*> 0*.*05*	*> 0*.*05*	
**Force-time integral (BW/ms)**	Acute PF (1)	77.51±19.22	38.43±9.38	168.05±41.15	0.22 ^(1–2)^ (small)
	Chronic PF (2)	74.01±14.71	48.87±16.24	208.29±60.36	0.79 ^(1–3)^ (large)
	Control (3)	64.40±14.09	46.27±11.75	192.96±27.14	0.69 ^(2–3)^ (medium)
	***p*** ^&^ ***-value***	*0*.*718* ^*(1–2)*^	*> 0*.*05*	*> 0*.*05*	
		*0*.*045* ^*(1–3)*^	*> 0*.*05*	*> 0*.*05*	
		*0*.*040* ^*(2–3)*^	*> 0*.*05*	*> 0*.*05*	
**Contact area (cm** ^**2**^ **)**	Acute PF (1)	36.6±3.9	45.0± 5.9	65.3±5.8	0.45 ^(1–2)^ (medium)
	Chronic PF (2)	34.7±5.1	42.5± 7.8	65.2±6.5	1.00 ^(1–3)^ (large)
	Control (3)	40.3±3.6	44.5± 5.2	67.1± 5.5	1.45 ^(2–3)^ (large)
	***p*** ^&^ ***-value***		*> 0*.*05*	*> 0*.*05*	
			*> 0*.*05*	*> 0*.*05*	
			*> 0*.*05*	*> 0*.*05*	
**Contact time (ms)**	Acute PF (1)	147.0± 16.9	182.8± 37.1	165.3± 25.2	0.38 ^(1–2)^ (small)
	Chronic PF (2)	151.0±16.1	179.2± 38.2	165.1±24.7	0.35 ^(1–3)^ (small)
	Control (3)	153.3±18.1	196.9± 34.1	175.1±24.0	0.13 ^(2–3)^ (small)
	***p*** ^&^ ***-value***	*> 0*.*05*	*> 0*.*05*	*> 0*.*05*	

^*&*^ *p* value were calculated using ANOVAS test and *Newman-Keuls post-hoc test*.

**Table 4:** Descriptive statistics (mean ± standard deviation) and comparisons between acute plantar fasciitis (PF), chronic PF and control groups regarding their total contact time of the foot.

Variable	Acute PF (1)	Chronic PF (2)	Control (3)	Value *p* ^&^
**Total Contact time (ms)**	230.4± 27.9	239.6± 24.5	234.0± 21.3	0.842 ^(1–2)^
				0.894 ^(1–3)^
				0.933 ^(2–3)^

^*&*^ *p* value were calculated using ANOVAS test and *Newman-Keuls post-hoc test*.

## Discussion

The main findings of this study confirmed the first and the second hypotheses, and showed that runners with PF presented higher dynamic plantar loads over the rearfoot compared to controls, regardless of the stage of the injury (chronic and acute), and, among runners with PF, those in the chronic stage showed higher plantar loads compared to those in the acute stage. However, different from what was expected for the third hypothesis, runners in the acute stage did not show increased plantar loads over the midfoot and forefoot, but over the calcaneal area, as expected by the typical pathophysiology development of PF. This was different from what has been observed in studies of plantar fasciitis during gait, in which the rate of plantar load remained in areas such as the forefoot [10, 11], midfoot [9], and toes [10, 11].

In the present study, the fact that during running the plantar loading rate was higher over the rearfoot (calcaneus) in the chronic stage of PF compared to both controls and runners in the acute stage of PF, may account for previous physiological results in subjects with PF. The loss of elasticity of the heel pad due to deposit fibrosis lead to a failure in the shock-absorbing mechanism, which resulted in higher loads over the rearfoot, as observed in the present study, followed by degeneration of the plantar fascia [13, 22, 24–26].

The result of a higher loading rate (20–80%) in runners with PF are in agreement with results reported by Pohl *et al*. (2009) [6], who also found higher loading rate (20–80%) during running in women with PF without pain (chronic stage). In addition to the reported results, the present study demonstrated that in the presence of pain (acute PF group), the plantar loading rate (20–80%) was lower compared to runners without pain (chronic PF group). The former findings suggested that the presence of pain symptom in the rearfoot (acute stages of PF) could lead to antalgic mechanisms that reduce plantar loads in the calcaneous area.

The plantar fascia elasticity was reduced in individuals with pain when compared to individuals without pain in Sahin’s study [27]. In addition, the pain associated with acute inflammation of the plantar fascia increases its thickness, which in turn decreases its capacity to support plantar loads [28, 29]. This morphological change of the plantar fascia generates an increase in the stretching tension of the fascia tissue during dynamic activities, and runners may adapt their running pattern by shortening the foot contact to the ground to avoid pain. These dynamic adaptations in runners with pain can be considered an antalgic protective mechanism that results in lower plantar loading over the rearfoot, as we observed in runners with acute PF in the present study.

However, in the chronic stage of PF there are different degenerative changes in the foot tissues compared to what happened in the acute stage. Degeneration and fibrosis of the plantar fascia, reduction in its thickness, and atrophy of the intrinsic foot muscles were observed in individuals in the chronic stage of PF [30]. These chronic tissue changes in runners without pain may lead to a withdrawal of the antalgic protective mechanism, resulting in higher plantar loads over the rearfoot, as observed in runners in the chronic stage compared to the acute PF and control groups. An appropriate plantar loading distribution during running is also a result of the cushioning properties of the footwear and the integrity of the heel tissues [31]; we standardized the type of running shoe used for all individuals assessed in the present study to minimize the footwear influence in the investigation of the injury stages. A limitation of this study was the estimation of the loading rate using a plantar pressure system with a sampling rate of 100Hz. The differences observed between runners with chronic PF and controls in the present study were also found in a previous study that calculated the loading rate using force plates at a 1000Hz [6].

These findings are clinically important for promoting better therapeutic protocols for runners with PF in the plantar loading reduction strategies during running practice. These therapeutic strategies to reduce plantar loading in runners has already been used with success in individuals with previous stress fractures [32]. The present results have the potential to improve therapeutics during different PF stages (acute and chronic) [33], especially in relation to recommendations for orthotics and insoles [34, 35].

## Conclusions

Runners with PF showed different dynamic patterns of plantar loads over the rearfoot area depending on which stage of the injury they were experiencing, but plantar load was always higher for the PF group than for the control runners. In the acute stage of PF, runners presented a lower loading rate and forces over the rearfoot area, possibly due to dynamic mechanisms of plantar fascia during running related to pain protection of the calcaneal area.
